# Impact of marine processes on flow dynamics of northern Antarctic Peninsula outlet glaciers

**DOI:** 10.1038/s41467-020-16658-y

**Published:** 2020-06-11

**Authors:** Helmut Rott, Jan Wuite, Jan De Rydt, G. Hilmar Gudmundsson, Dana Floricioiu, Wolfgang Rack

**Affiliations:** 1 ENVEO IT GmbH, 6020 Innsbruck, Austria; 2 Institute of Atmospheric and Cryospheric Sciences, University of Innsbruck, 6020 Innsbruck, Austria; 3 Department of Geography and Environmental Sciences, Northumbria University, Newcastle upon Tyne, NE1 8ST UK; 4 Institute for Remote Sensing Technology, German Aerospace Center, Oberpfaffenhofen, 82234 Wessling, Germany; 5 Gateway Antarctica, University of Canterbury, Christchurch, 8140 New Zealand

**Keywords:** Climate sciences, Cryospheric science

**Arising from** P. A. Tuckett et al., *Nature Communications* 10.1038/s41467-019-12039-2 (2019).

Tuckett et al.^1^ report on short-term events of ice flow acceleration on five outlet glaciers of the northern Antarctic Peninsula and their relation to numerical model output of surface melt. The authors argue that the delivery of surface meltwater to the glacier bed transiently increases the basal water pressure and enhances basal motion, causing near-instantaneous flow acceleration followed by subsequent drainage causing deceleration. An outdated version of the grounding line (GL) vector, separating grounded and floating glacier ice, is used so that the majority of the analyzed velocity points are located on floating sections of glacier termini where meltwater drainage does not have any effect on subglacial water pressure. Our independent analysis of glacier surface velocities exhibits for the speed-up events only marginal changes in velocity on grounded ice and a significant increase of velocity on floating glacier sections progressing on prefrontal sea ice and ice mélange, clear evidence for the dominant influence of ocean dynamic forcing as previously reported by refs. ^2–4^.

The main data products used in ref. ^1^ are time series of 6-day mean velocities between October 2016 and April 2018 for glaciers discharging into the Larsen A embayment (Drygalski Glacier), the Larsen B embayment (Crane, Hektoria, Jorum glaciers), and the Gerlache Strait (Cayley Glacier). Velocities are derived from 6-day repeat-pass radar satellite images of the Sentinel-1 mission, aggregated into 1 km squared boxes at distances 1 km up to 10 km upstream of the glacier fronts. The interpretation of the velocity data in terms of ice flow dynamics is critically based on the assumption that the boxes are located on grounded ice. However, high-resolution digital elevation models (DEMs) of the TanDEM-X satellite mission, acquired in mid-2011, −2013, −2016 and the analysis of surface elevation change (SEC) show that major sections were afloat already in these years^4^. The authors used an outdated GL version, so that in total 24 out of the 36 velocity boxes shown are located on floating sections of glacier termini: all 10 velocity boxes on Hektoria Glacier, all six velocity boxes on Crane Glacier, three boxes on both Drygalski and Jorum glaciers and two boxes on Cayley Glacier.

In Fig. 1, we show the GL locations of Hektoria Glacier in 2013 and 2016, based on the break in slope in DEMs and on the SEC (Supplementary Figure 2). The glacier front advanced by 12 km between 2011 and 2016. The floating section of the terminus covered in 2016 an area of 135 km^2^. The persistent sea ice cover since mid-2011 in the proglacial bay impeded calving, leading to terminus advance whilst glacier thinning on grounded ice continued. Whereas the termini of Hektoria and Green glaciers extend into a wide bay, the tongues of the other studied glaciers are narrower and laterally confined by mountain ridges. The floating parts of these glaciers extend several kilometers inland of the front along the center of the terminus, whereas the lateral margins rest on slopes, as shown in Fig. 2d for Crane Glacier.

**Fig. 1:**
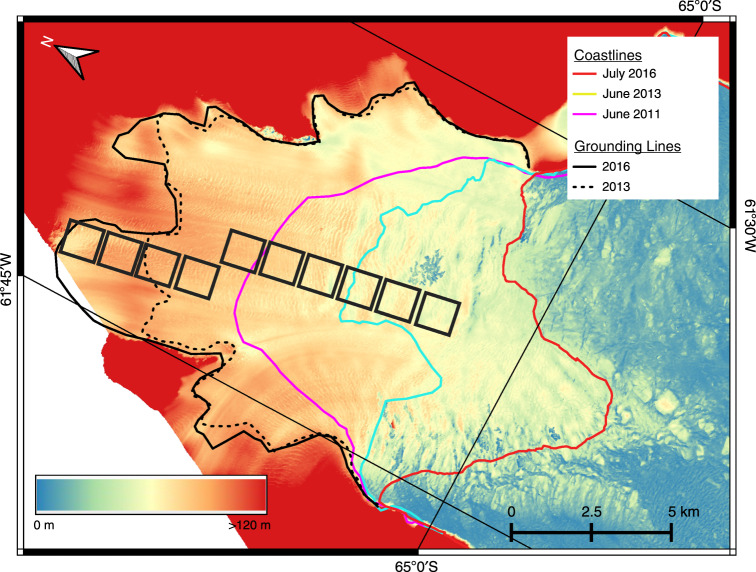
Extent of floating ice on Hektoria Glacier terminus. TanDEM-X DEM of Hektoria and Green glacier terminus, 2016-07-27, with coastlines in June 2011, June 2013 and July 2016, grounding lines (GL) in June 2013 and July 2016. Colour code for altitude from 0 to ≥120 m a.s.l. The GL location is based on break in slope in the DEM and maps of surface elevation change 2013–2016^4^. The black boxes show the sites of the velocity data of ref. ^1^.

**Fig. 2:**
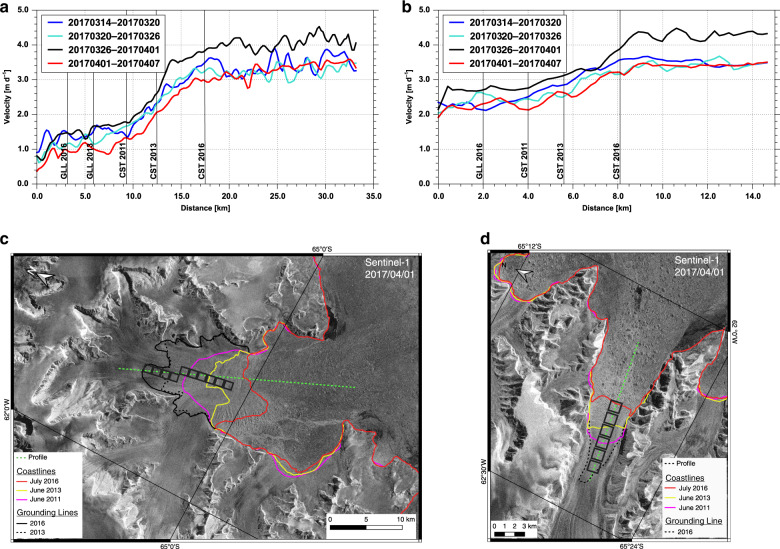
Velocity transects during a down-slope wind event. Velocity transects along central flowlines, extending from grounded ice to floating ice and ice mélange for Hektoria Glacier (**a**) and Crane Glacier (**b**) before, during and after the acceleration event of 2017-03-26 to 2017-04-01. GLL – Location of the grounding line; CST – glacier front in 2011, 2013, 2016. **c** and **d**: Sections of Sentinel-1 image of 2017-04-01 showing the location of the velocity transects on Hektoria Glacier (**c**) and Crane Glacier (**d**).

We generated ice velocity maps of Antarctic outlet glaciers since 2015, in 12- and 6-day time intervals, depending on data availability according to the Sentinel-1 operation plan^5^. Here, we argue that the velocity time series provide clear evidence for the dominant impact of oceanic forcing during the acceleration events highlighted by ref. ^1^. The examples in Fig. 2 show a distinct increase in the velocity magnitude along the central flowlines of Hektoria and Crane glaciers from grounded ice across the floating terminus and further on in the prefrontal ice mélange and sea ice. In the absence of a solid sea ice cover (as for Cayley and Drygalski glaciers), the tracking of bergy bits and ice mélange provides estimates on prefrontal drift velocities. The impact of strong off-shore winds and ocean currents during acceleration events is also evident in Sentinel-1 amplitude images, showing fast off-coast movement of ice mélange. For example, the plume of ice touching the front of Drygalski Glacier on 21 March 2018 had been displaced 20 km eastward by 27 March 2018 (Supplementary Figure 3). Removal of ice mélange causes significant short-term acceleration of marine-terminating glaciers^6^. Over the same time span, the young sea ice in front of the multiyear pack ice in the Larsen B embayment drifted eastward by 15 km. Oceanic processes also have a large impact on multiannual variations of flow velocity and mass balance. Persistent multiannual sea ice in the Larsen A embayment (from mid-2013 to 2016) and in the Larsen B embayment (since winter 2011) caused major decrease of flow velocities and the mass losses of grounded ice dropped from 9.73 Gt a^−1^ during 2011–2013 to 4.70 Gt a^−1^ during 2013–2016^4^.

A further point of concern is the use of relative changes in velocity in ref. ^1^ as a basis for inferring conclusions about the sources for speed-up. First, it is unclear which criteria are used for selecting the melt-induced acceleration events out of the full sample of velocity spikes coinciding with modeled surface melt. Second, the authors claim that a larger relative increase in velocity closer to the glacier front would be needed if marine processes were the trigger for the speed-up. Beside this claim being based on relative velocity change, whereas any flux and mass considerations must be based on actual velocities, no quantitative analysis is provided to support this statement. In addition, the assumption that surface meltwater of the transient events finds its way to the glacier bed and causes an increase of subglacial water pressure is speculative and not relevant for floating ice. Apart from melt intensity, the freezing state of the snow/firn/ice body needs to be taken into account. The Larsen outlet glaciers have cold snow/ice bodies in which a substantial portion of the meltwater released at the surface would freeze, in particular after cold periods^7^. Estimates on the intensity and spatial extent of melt events can be deduced from C-band backscatter signatures^8^. For example, the event in March 2018 shows a modest decrease of the backscatter intensity on only one date (27 March 2018), indicating a short period of modest surface melt not able to release sufficient water for drainage to the glacier bed.

Another critical issue is the neglect of biases in the retrieved velocities caused by shifts in the radar line-of-sight (LOS) distance by several meters owing to changes in radar signal penetration associated with change from dry to wet snow and vice versa. Depending on the flow direction relative to LOS, this shift causes an underestimation or overestimation of the velocity (Supplementary Note 1). The Sentinel-1 data used in the study are from descending orbits so that the transition from dry to wet snow introduces a decrease of velocity for glaciers, heading west and an increase for glaciers heading east, as evident in the different timing of apparent acceleration on Cayley and Drygalski glaciers (Supplementary Figure 1).

The issues addressed above question the interpretation of the presented material in terms of melt-induced acceleration. Our analysis of velocity time series on grounded and floating glacier sections and the motion of proglacial sea ice and ice mélange confirm the findings of previous publications that changes in velocity and ice export of northern Antarctic Peninsula outlet glaciers during recent years have been primarily governed by frontal stress perturbations propagating up-glacier and by variations in oceanic boundary conditions.

## Supplementary information

Supplementary Information

Description of Additional Supplementary Files

Supplementary Data 1

Supplementary Data 2

## Data Availability

Data generated during the study are included in the supplementary information files. Specifications and public availability of additional data (surface topography and ice velocity) generated for previous studies and used in support of this study are detailed in ref. ^4^.
