# Supplementary material for: Impact of marine processes on flow dynamics of northern Antarctic Peninsula outlet glaciers
Source: Nat Commun. 2020 Jun 11;11:2969. doi: 10.1038/s41467-020-16658-y (PMC7289832; doi:10.1038/s41467-020-16658-y)
Supplement: Supplementary file 1 — Supplementary Information [file 41467_2020_16658_MOESM1_ESM.docx]

Supplementary Information for

**Impact of marine processes on flow dynamics of northern Antarctic Peninsula outlet glaciers**

Rott, H.^1,2 *^, Wuite, J.^1^, De Rydt, J.^3^, Gudmundsson, G.H.^3^, Floricioiu, D.^4^, Rack, W.^5^

Affiliation:

^1^ENVEO IT GmbH, 6020 Innsbruck, Austria

^2^Institute of Atmospheric and Cryospheric Sciences, University of Innsbruck, 6020 Innsbruck, Austria

^3^Department of Geography and Environmental Sciences, Northumbria University, Newcastle upon Tyne, NE1 8ST, UK

^4^Institute for Remote Sensing Technology, German Aerospace Center, Oberpfaffenhofen, 82234 Wessling, Germany

^5^Gateway Antarctica, University of Canterbury, Christchurch 8140, New Zealand

*Correspondence to: [Helmut.Rott@enveo.at](mailto:Helmut.Rott@enveo.at)

# Supplementary Notes

**Supplementary Note 1:** ***Bias in 2D glacier surface velocity due to changes in radar signal penetration and impact of random noise***

The velocity time series in Figures 2 and 3 and Supplementary Figures 1 to 4 of Tuckett et al. [1] show temporal patterns (acceleration/deceleration) reflecting the 6-day repeat observation interval of the Sentinel-1 data used for the velocity retrievals by means of offset tracking. The authors report a median velocity error of 30.5 m/yr (corresponding to 0.5 m for a 6-day time span) based on the mean displacement over bedrock areas. The actual error for individual time spans, accounting for bias and random errors, can deviate on glaciers significantly from this estimate, as explained below.

Offset tracking of SAR data from a single swath delivers only two components of the velocity vector, the displacements in azimuth, d_AZ_, and the displacement in line-of-sight, d_LOS_. For a right-looking radar system the relations between d_AZ_ and d_LOS_ and the 3D displacement vector are:

$$d_{AZ}= d_{E}\sin\alpha+ d_{N}\cos\alpha$$

$$d_{LOS}= d_{E}sin \theta cos \alpha- d_{N}sin \theta sin \alpha-d_{U}\cos\theta$$

θ - incidence angle of the radar beam; α - heading direction of the satellite (positive clockwise from north); [d_E_, d_N_, d_U_]^T^ – offsets in east, north, vertical direction. The signs in the equations use the common convention that displacement towards the sensor yields negative values and displacement away positive values. The 2D retrieval, performed in azimuth and line-of-sight (LOS), assumes stable surface (d_U_ = 0). This means that a change in elevation of the source of the reflected radar signal (the location of the scattering phase centre, φ_s_) yields a bias in the retrieved velocity, the magnitude of which depends on the shift d_U_, the incidence angle and the angular difference between α and the flow direction of the glacier.

The velocity time series of [1] includes several 6-day time spans with changes from dry to melting snow/firn and vice versa. Retrieved velocity data of these events are (partly) impaired by a bias due to temporal variations in the location of the scattering phase centre in the snow/firn medium related to changes in signal penetration. On the northern Antarctic Peninsula the heading angle α is 210° for Sentinel-1 descending orbit images. This means a bias related to d_U_ is zero for a glacier moving NNE or SSW, and maximum for a glacier moving towards WNW (300°) or ESE (120°). A shift of the φ_s_ – position towards the sensor (due to switchover from dry to wet snow) yields a maximum decrease in displacement for a glacier heading towards WNW and a maximum increase for a glacier heading towards ESE. As this bias does not depend on the velocity magnitude, the relative error (resembling an acceleration respectively deceleration signal) increases with decreasing flow velocity.

Changes from dry to wet snow introduce shifts in the radar LOS distance up to several metres. Rizzoli et al. [2] report for frozen firn in the percolation zone of Greenland an average X-band (vertical) penetration bias of 3.9 m. Measurements on the East Antarctic Plateau and in the coastal percolation zone show higher values of signal penetration for C-band [3], as expected according to theory. For fine grained snow the C-band 2-way penetration length (corresponding to the LOS shift of φ_s_ vs. the actual surface) is >10 m and for the percolation zone in the order of 5 m. The actual penetration bias for a cold snow/firn medium depends on the volume scattering properties which are related to the microstructure. The C-band backscattering signal of wet snow is controlled by scattering at the air/snow interface whereas dry seasonal snow is easily penetrated [4]. The backscatter signal of glacier ice and sea ice arises from the surface or near surface layers.

The opposite behaviour of acceleration/deceleration on the east and west coast glaciers during the period 3 to 15 Nov. 2017 (high-lighted in Fig. 3 of [1]) is very likely an artefact caused by a change of radar signal penetration. The σ°-values in the firn area of Cayley Glacier on 3, 9, 15 Nov. are -4 dB, -17 dB, -8 dB, on Drygalski Glacier -3 dB, -14 dB, -9 dB indicating a temporal sequence of completely frozen snow/firn, surface melt, partial refreezing. For 3 to 9 Nov. (associated with a modelled melt event) the change from dry to wet snow coincides with an apparent acceleration signal on Drygalski Glacier and an apparent deceleration signal on Cayley Glacier (Supplementary Figure 1).

Whereas changes in radar signal penetration are causing biases in the retrieved velocities, the 6-day pattern throughout the Sentinel-1 velocity time series suggests also significant impacts of random noise related to the performance of image template cross-correlation for which the quality and stability of surface features are critical. Epochs with changes in σ° due to alteration between dry and wet snow conditions are changing the features to be tracked, resulting in increased random errors.

In situ velocity time series, measured at two permanent GPS stations on Flask Glacier, do not show such a pattern [5]. Flask Glacier is located approximately 30 km south of Crane Glacier and drains into the remnant part of Larsen B Ice Shelf in SCAR Inlet. From February 2011 to November 2012 the station GPS 1 operated 20 km and the station GPS 2 15 km upstream of the grounding line. Both stations show very stable velocities. At GPS 2 the mean velocity was 266.8 m/yr and the overall deviation from the mean value varied at maximum by ± 0.6 m over the whole epoch. Sentinel-1 6- and 12-day velocity time series, available for Flask Glacier since 2014, show much higher temporal variability, very likely due to random noise as to be expected for template matching of image data with 5 m x 20 m spatial resolution over confined glaciers and short time intervals. Although the Sentinel-1 velocity data of Flask Glacier are not from the same epoch, the mean velocities are similar to those of the GPS epoch so that major changes in the temporal pattern are unlikely. Consequently, possible impacts of a bias due to changing radar signal penetration and random noise should be taken into consideration for the interpretation of the Sentinel-1 velocity time series.

.

# Supplementary Figures

**
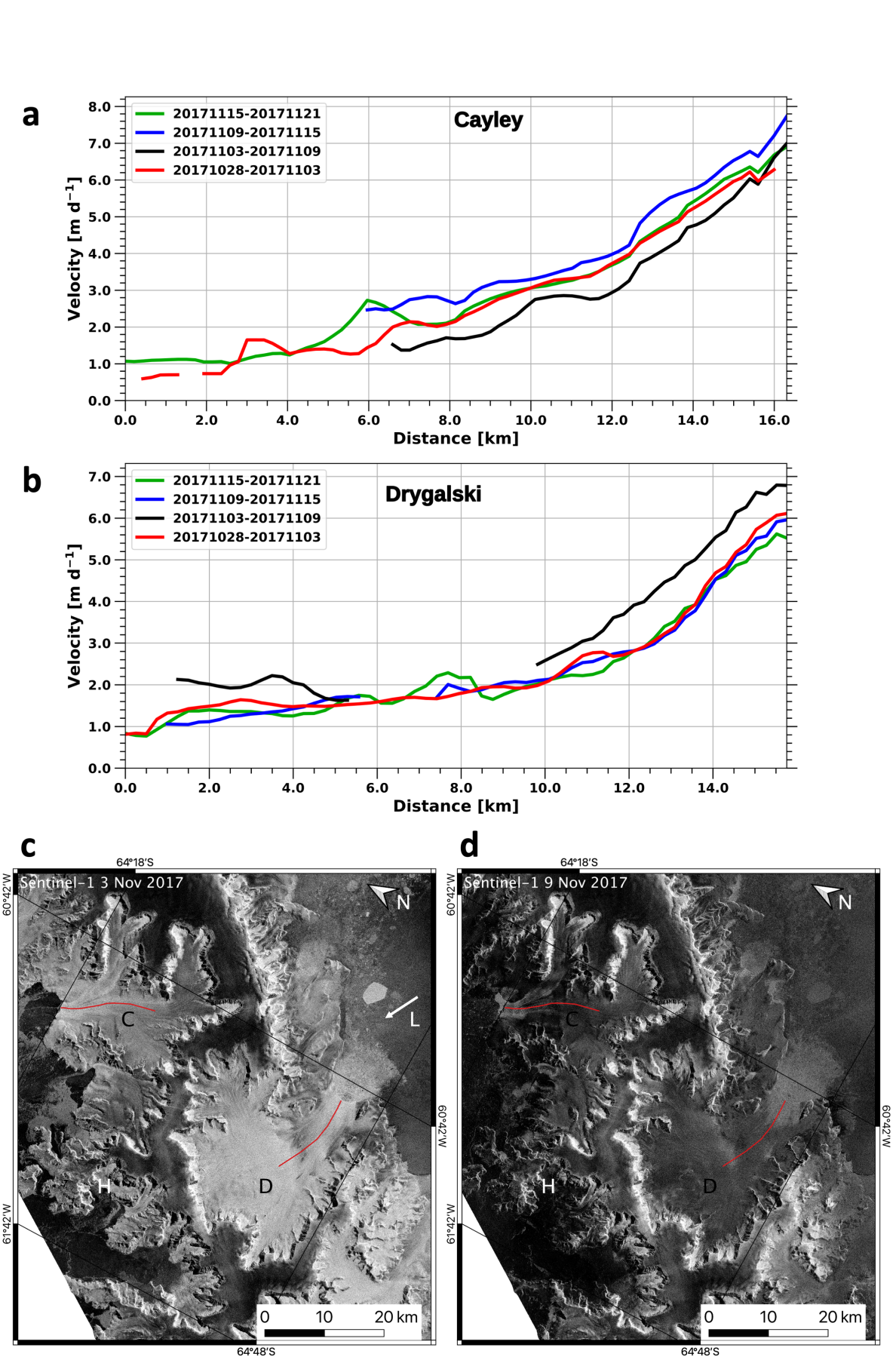
**

**Supplementary Figure 1.** **Impact of change from dry to melting snow on flow velocity**. Transects of Sentinel-1 velocities along the central flowline of (**a**) Cayley and (**b**) Drygalski glaciers (front at 0 km). Sentinel-1 backscatter images of Cayley (C) and Drygalski (D) glaciers on (**c**) 3 November 2017, (**d**) 9 November 2017, with the location of the velocity transects. L –radar look direction. The switch from high to low backscatter intensity from 3 to 9 November is caused by transition from dry to melting snow.

**
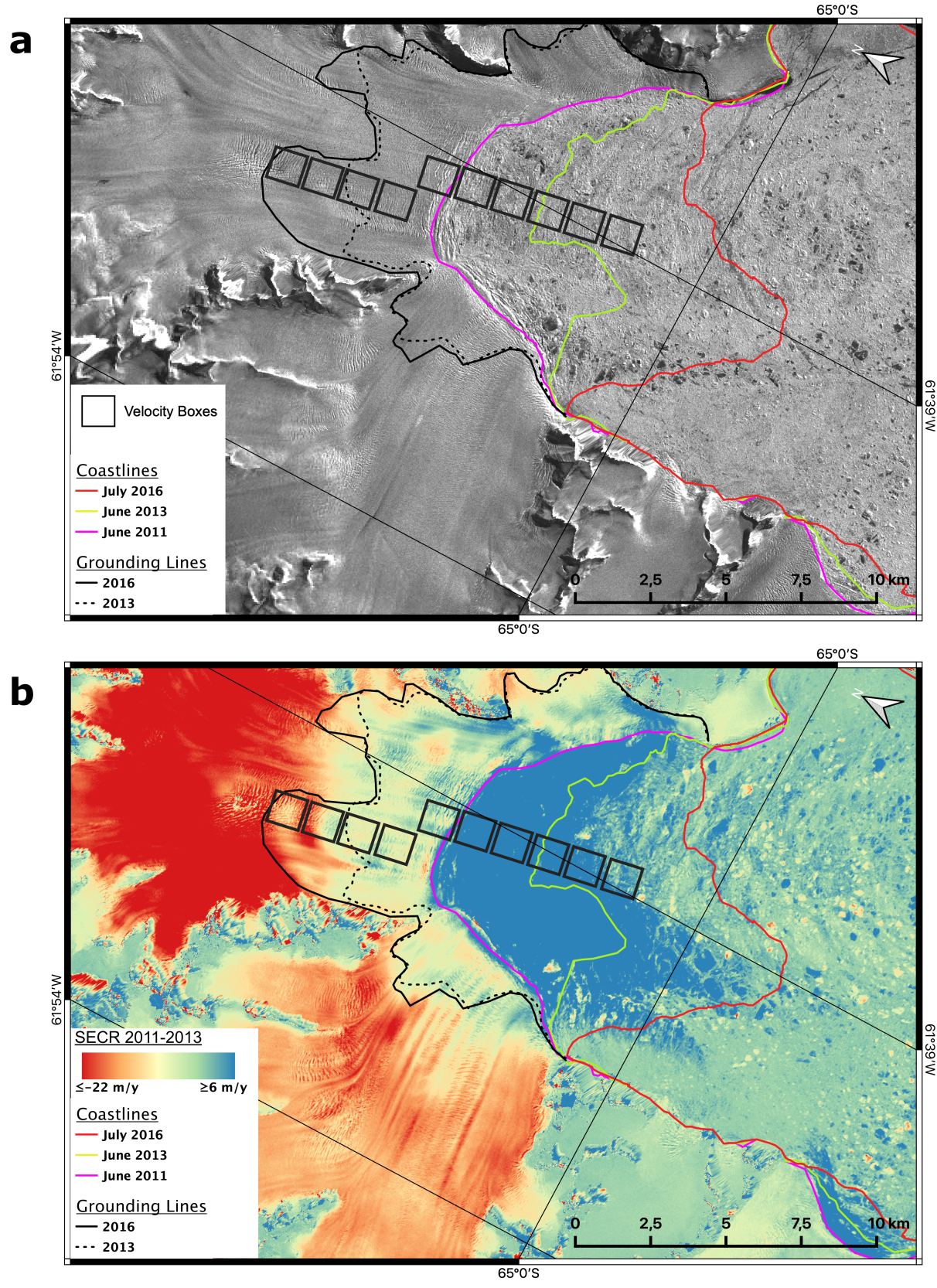
**

**Supplementary Figure 2**. (**a)** TanDEM-X amplitude image of Hektoria and Green glacier terminus, 2011-06-25, with coastlines in June 2011, June 2013 and July 2016 and grounding lines in June 2013 and July 2016. The black boxes show the sites of the velocity data of Tuckett et al. [1]. **(b)** Map of rate of surface elevation change (dh/dt, m/yr) from June 2011 to June 2013 based on the elevation difference in TanDEM-X DEMs. Colour code from ≤-22 m/yr to ≥+6 m/yr. Details on the data base and method in [6].

**
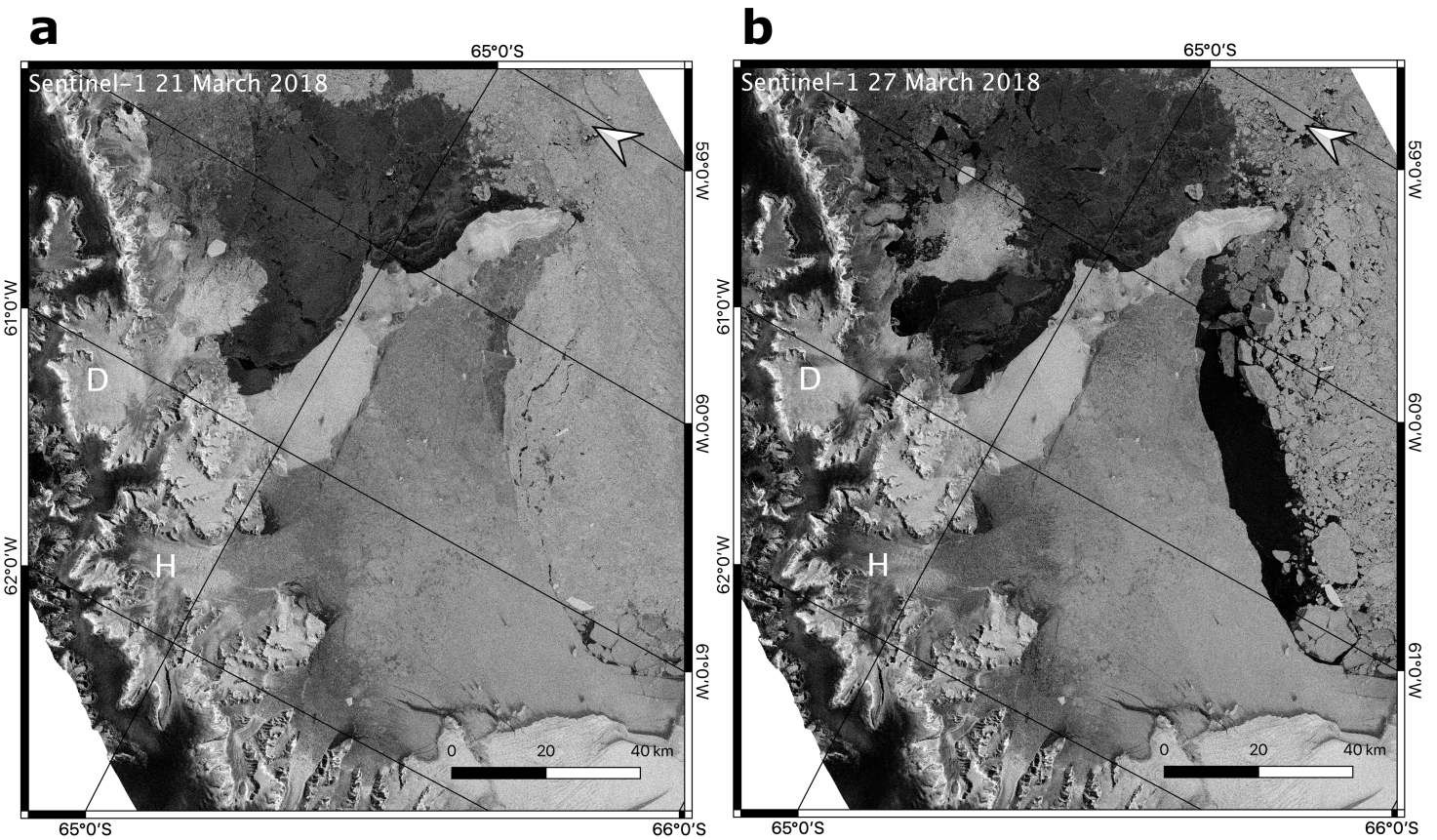
**

**Supplementary Figure 3.** **Off-coast drift of floating ice.** Sentinel-1 amplitude images, Larsen A and B coast on (**a)** 21 March 2018, **(b)** 27 March 2018*,* showing the rapid off-coast drift of ice mélange in front of Drygalski Glacier (D), break up and drift of the fractured sea ice east of the compact sea ice cover in Larsen B embayment, and further opening of a costal polynya north-east of the Hektoria Glacier front.

**Supplementary References**

1. Tuckett, P.A. et al. Rapid accelerations of Antarctic Peninsula outlet glaciers driven by surface melt. *Nat. Commun.* **10**, 4311, https://doi.org/10.1038/s41467-019-12039-2 (2019).
2. Rizzoli, P. et al. Characterization of snow facies on the Greenland Ice Sheet observed by TanDEM-X interferometric SAR data. *Remote Sens.* **9**(4), 315; doi:[10.3390/rs9040315](http://dx.doi.org/10.3390/rs9040315) (2017).
3. Rott H. et al. Active and passive microwave signatures of Antarctic firn by means of field measurements and satellite data. *Ann. Glaciol.* **17**, 337-343 (1993).
4. Ulaby, F.T. et al. Microwave Radar and Radiometric Remote Sensing. Ann Arbor: University of Michigan Press (2014).
5. Farinotti, D. et al. The ice thickness distribution of Flask Glacier, Antarctic Peninsula, determined by combining radio-echo soundings, surface velocity data and ﬂow modelling. *Ann. Glaciol.* **54**, 18–24, doi:10.3189/2013AoG63A603 (2013).
6. Rott, H. et al. Changing pattern of ice ﬂow and mass balance for glaciers discharging into the Larsen A and B embayments, Antarctic Peninsula, 2011 to 2016. *Cryosphere* **12**, 1273–1291, https://doi.org/10.5194/tc-12-1273-2018 (2018).
