# Supplementary material for: Impact of marine processes on flow dynamics of northern Antarctic Peninsula outlet glaciers
Source: Nat Commun. 2020 Jun 11;11:2969. doi: 10.1038/s41467-020-16658-y (PMC7289832; doi:10.1038/s41467-020-16658-y)
Supplement: Supplementary file 2 — Description of Additional Supplementary Files [file 41467_2020_16658_MOESM2_ESM.docx]

**Description of Additional Supplementary Files**

**File Name**: Supplementary Data 1

**Description**: Geographic location (Antarctic Polar Stereographic Projection) and magnitude of glacier surface velocity for the transects shown in Figure 2a (Hektoria Glacier), Figure 2b (Crane Glacier), Supplementary Figure 1a (Cayley Glacier), Supplementary Figure 1b (Drygalski Glacier).

**File Name**: Supplementary Data 2

**Description**: Geographic location of coastlines of northern Larsen-B embayment, Antarctic Peninsula, (2011, 2013, 2016) including the fronts of Hektoria Glacier and Crane Glacier and grounding lines of Hektoria Glacier (2013, 2016) and Crane Glacier (2016)
